# Dynamic Epigenetic Control of Highly Conserved Noncoding Elements

**DOI:** 10.1371/journal.pone.0109326

**Published:** 2014-10-07

**Authors:** Loqmane Seridi, Taewoo Ryu, Timothy Ravasi

**Affiliations:** 1 Division of Biological and Environmental Sciences & Engineering, Division of Applied Mathematics and Computer Sciences, King Abdullah University of Science and Technology, Thuwal, Kingdom of Saudi Arabia; 2 Department of Medicine, Division of Genetics, University of California San Diego, La Jolla, California, United States of America; CNRS UMR7622 & University Paris 6 Pierre-et-Marie-Curie, France

## Abstract

**Background:**

Many noncoding genomic loci have remained constant over long evolutionary periods, suggesting that they are exposed to strong selective pressures. The molecular functions of these elements have been partially elucidated, but the fundamental reason for their extreme conservation is still unknown.

**Results:**

To gain new insights into the extreme selection of highly conserved noncoding elements (HCNEs), we used a systematic analysis of multi-omic data to study the epigenetic regulation of such elements during the development of *Drosophila melanogaster*. At the sequence level, HCNEs are GC-rich and have a characteristic oligomeric composition. They have higher levels of stable nucleosome occupancy than their flanking regions, and lower levels of mononucleosomes and H3.3, suggesting that these regions reside in compact chromatin. Furthermore, these regions showed remarkable modulations in histone modification and the expression levels of adjacent genes during development. Although HCNEs are primarily initiated late in replication, about 10% were related to early replication origins. Finally, HCNEs showed strong enrichment within lamina-associated domains.

**Conclusion:**

HCNEs have distinct and protective sequence properties, undergo dynamic epigenetic regulation, and appear to be associated with the structural components of the chromatin, replication origins, and nuclear matrix. These observations indicate that such elements are likely to have essential cellular functions, and offer insights into their epigenetic properties.

## Introduction

Genomic DNA is subject to diverse mutations caused by chemicals, replication errors, and mobile genetic elements. Coding sequences are generally under higher selective pressure than noncoding sequences, due to the essential roles that proteins play in the cell [1]. However, some noncoding regions show extreme conservation (even more than coding sequences) over very long evolutionary timeframes [2], [3]. These extremely conserved sequences are found universally in multicellular eukaryotes of the animal and plant kingdoms [3], [4], indicating that such sequences have essential functions.

Indeed, many highly conserved noncoding elements (HCNEs), noncoding loci that maintain high level of similarity across different species, function as developmental enhancers [5], enhancer-blocking insulators [6], and regulators of splicing [7] and RNA editing [8]. Mutations in HCNEs have been associated with various diseases, including cancers and neurodevelopmental disorders [9]–[11]. However, these functions of HCNEs reflect the activities of the DNA-interacting proteins that bind to very short and degenerate DNA sequences within them [12], and are insufficient to explain the invariability of HCNEs (≥200 bp) during evolution. Even the enhanceosome model, which requires a strict array of transcription factor (TF) binding sites over a long sequence [13], does not explain the observed sequence conservation between the TF binding sites. We therefore speculated that HCNEs are subjected to a higher level of protection against mutations compared to other sequences.

In eukaryotic cells, histones pack DNA into nucleosomes to form the chromatin structure; this protects DNA from damage, and offers an additional layer of regulation via the chemical modification of histones [14], [15]. A few previous studies have suggested that HCNEs may be associated with epigenetic control mechanisms. For instance, an analysis of mammalian stem cells found epigenetic modifications of some HCNEs, such as the bivalent methylation, H3K27me3 + H3K4me3 [14], [16], [17]. In addition, 11% of mammalian HCNEs co-occur with scaffold/matrix attachment regions (S/MARs), which have been implicated in the structural organization and remodeling of chromatin [18]. To understand the properties underlying the extreme conservation of HCNEs, however, we need data from a systematic analysis of their potential epigenetic regulation.

Here, we performed an integrative analysis of the epigenetic properties and regulations of HCNEs throughout the development of *Drosophila melanogaster*. Our results indicate the following: HCNEs intrinsically favor stable nucleosome occupancy at the sequence level; HCNEs reside within nucleosome-enriched and mononucleosome- and H3.3-depleted regions in S2 cells; the chromatin regions around HCNEs undergo significant changes in epigenetic modification during development, and such changes are correlated with the transcription levels of flanking genes; most HCNEs fire later in replication, however some serve as early replication origins; and HCNEs are significantly associated with lamina-associated domains (LADs). Our results collectively indicate that HCNEs are under special evolutionary control at the levels of chromatin and nuclear structural organization.

## Results

### HCNEs in the *D. melanogaster* genome

Using a minimum average conservation score of 0.95 across 14 insect species that diverged 2.3 to 366 million years ago [19], we identified 1,456 HCNEs ≥200 bp in the *D. melanogaster* genome (Table S1). Their level of conservation was greater than that of the protein-coding sequences in this genome (Figure S1). More than half of the HCNEs (56.94%) were intergenic, while the rest were intronic.

**Figure 1:**
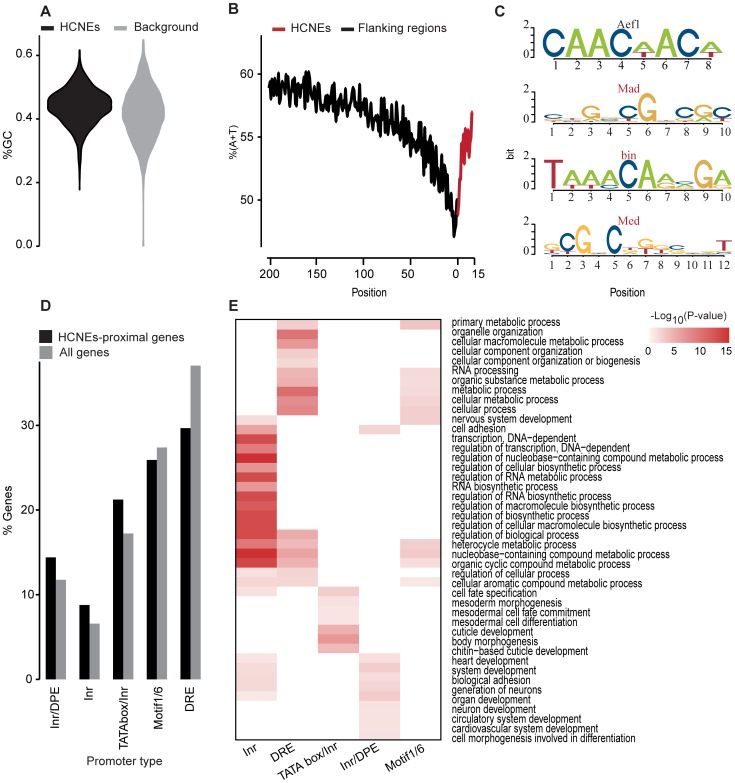
Genomic properties of HCNEs. (A) Violin plot illustrating that HCNEs have a higher GC content versus 10,000 random non-coding sequences. (B) Per-base A+T frequencies within 200 bp of HCNE-flanking regions and 15 bp of HCNEs aligned at their boundaries (region on downstream boundaries were reversed). (C) Sequence logos of four TFBSs that were among the most significant matches to overrepresented heptamers; red marks TFs reported to be involved in developmental processes; for complete list of TFBSs matches to top 50 overrepresented heptamers refer to Table S2. (D) Bar plot showing the fraction of core promoter predictions among the HCNE-proximal genes. HCNE-proximal genes are enriched in the Inr-motif and depleted of Motif1/6 and DRE core promoters, to a higher degree than expected by chance. (E) GO enrichment among HCNE-proximal genes grouped by their core promoter type. The top 10 significantly enriched terms (Holm-Bonferroni adjusted P-values <0.05) are shown for each promoter-type group. Colors represent –log_10_ (Holm-Bonferroni adjusted P-values).

The identified HCNEs displayed distinct sequence properties. They had higher GC contents (Figure 1A) (P = 2.29e-29; P-values were obtained using the Mann-Whitney test throughout, unless otherwise specified) compared to the random noncoding sequences. Moreover, the frequency of A and T nucleotides was found to drop sharply at the boundaries of HCNEs and increase smoothly in the surrounding regions (Figure 1B), in a pattern that is conserved across different lineages (including insects) [20], [21]. Interestingly, the central regions of the HCNEs were slightly GC-poor, similar to the sequences of short conserved elements [22] (data not shown).

To examine the intrinsic biological roles of the conserved regions, we queried the 50 most overrepresented heptamer against FlyReg motifs [23] using STAMP [24] and detected many putative TF binding sites (TFBSs) related to developmental TFs (Figure 1C and Table S2). This result is consistent with previous reports showing that HCNEs harbor binding sites for developmental TFs [3], [25].

Gene regulatory blocks (GRBs) are broad genomic regions of conserved synteny that harbor dense distributions of HCNE loci, developmental and regulatory genes. GRBs are thought to have emerged due to evolutionary pressure to maintain HCNEs and their target genes in *cis*, both in vertebrates and in insects [26], [27]. To assess the amount of overlap between the identified HCNEs and GRBs, we determined the GRB boundaries from the coordinates of 1,319 genes that show conserved microsynteny across the *Drosophila* genus [28] and span regions of dense HCNE loci [29]. We found that 51.44% of the identified HCNEs reside inside 110 GRBs, and an additional 7.35% lie within 50 kb of a GRB boundary.

HCNEs located inside GRBs are thought to regulate genes that show conserved microsynteny and are characterized by involvement in “regulation of transcription” and “multicellular-organismal development” [26]. Of them, 95% contain an initiator element (Inr) motif in their core promoter, consisting of Inr only, Inr followed by a downstream promoter element (Inr/DPE), or a TATA box followed by an initiator element (TATA box/Inr) [26]. However, 49% of insect GRBs do not contain any gene that satisfies these characteristics, suggesting that the criteria are either insufficient to characterize HCNE targets, or that regulation by HCNEs is not restricted to Inr-motif promoters [28]. Moreover, around 41% of the identified HCNEs were not associated with any GRB. Therefore, for downstream analysis, we included all genes lying within 50 kb from the boundary of a GRB or a HCNE (for HCNEs that were not located in close proximity to a GRB). These are henceforth referred to as “HCNE-proximal” genes. In this study, we focused on genes that had trustworthy promoter-type predictions available; *Drosophila* core promoters are classified into five types: Inr-only, Inr/DPE, TATA box/Inr, Motif1 followed by Motif6 (Motif1/6) (as described in [30]) and DNA replication element binding factor (DRE) core promoters [26], [30].

We identified 7,291 HCNE-proximal genes (approximately 39% of all annotated genes in *Drosophila* genome), 2,612 of which had reliable predictions available for their core promoter types. The distribution of core promoter types among the HCNE-proximal genes was significantly different from expected (P = 3.94e-5 by Chi-square homogeneity test; Figure 1D). The HCNE-proximal genes were prominently enriched in genes with Inr-motif promoters and depleted in genes Motif1/6 and DRE promoters.

Consistent with the previously detected differences in Gene Ontology (GO) enrichment between genes of distinct promoter types [26], the HCNE-proximal genes with Inr-only promoters were predominantly enriched in biological processes related to regulation and development, such as “regulation of transcription, DNA-dependent” and “system development”. Many GO terms related to developmental and cell adhesion processes were enriched among genes with Inr-only and Inr/DPE promoters. Meanwhile the genes with Mortif1/6 and DRE promoters tended to be involved in general processes such as “metabolic process” and “cellular process” (Figure 1E and Table S3). Interestingly, we detected some previously unreported differences that may reflect updates in the GO annotations. Most notably, genes with TATA box/Inr promoter were enriched in terms related to developmental processes, such as “cell fate specification” which was also enriched among genes with Inr-only promoter, and “mesodermal morphogenesis” and “cuticle development,” which were not enriched in the genes of other promoter types (Figure 1E and Table S3). Protein domain analysis suggests similar results for genes with Inr only and Inr/DPE promoters: genes with Inr-only promoter were enriched in homeobox protein domains; genes with Inr/DPE promoter were enriched immunoglobulin protein domains. However, genes with TATA box/Inr promoter were enriched in protein domains of unknown function and no protein domains were enriched among genes with Motif1/6 and DRE promoters (Table S4).

### Nucleosome landscape in the proximity of HCNEs

Nucleosome occupancy and positioning is intimately related to the regulation and protection of genetic material [15]. Nucleosomes occupy coding sequences more highly than intergenic regions [31]. Regions ∼150 bp upstream of transcriptional start sites (TSSs), which typically harbor many TFBSs, are generally depleted of nucleosomes [32]. Enrichment of nucleosomes in a promoter region is negatively correlated with gene expression [31].

**Figure 2:**
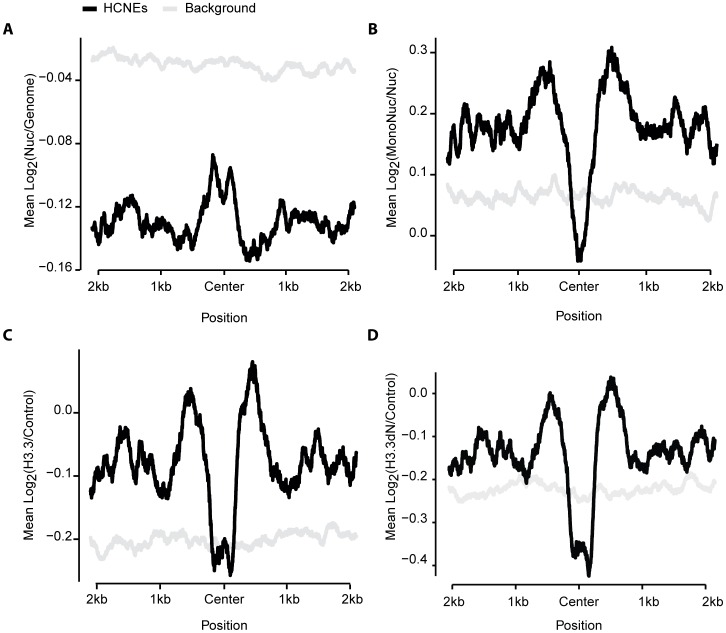
Nucleosome landscape at HCNEs. (A) Mean nucleosome density of sequences aligned with respect to their centers. Nucleosomes are enriched at the centers of HCNEs compared to the flanking regions. (B) Mononucleosome enrichment was calculated from sequences aligned as described in (A). Mononucleosomes are depleted in HCNEs compared to flanking regions. (C) H3.3 enrichment, calculated from sequences aligned as described in (A). H3.3 is depleted at HCNEs compared to the flanking regions. (D) Same as (C) but for H3.3dN.

To assess the intrinsic information embedded in HCNEs, we analyzed their nucleosome occupancy in the *D. melanogaster* embryonic S2 cell line [33]. We observed that the nucleosome density was higher in HCNEs compared to their flanking regions (Figure 2A) and slightly lower at the center of HCNEs. This pattern was similar to that previously reported for short HCNEs [22]. In addition, mononucleosomes were depleted at the centers of HCNEs, enriched at their borders and immediate flanking sequences, and showed smooth decreases along their distal flanking regions (Figure 2B).

H3.3, which is a non-canonical histone that replaces H3.1 during chromatin-disrupting processes, such as transcriptional regulation [34]–[37], has been shown to be important for fruit fly fertility and mammalian development [38], [39]. Therefore, we investigated the occupancy of H3.3 and H3.3dN (an N-terminal-region-lacking H3.3 that undergoes replication-independent incorporation into chromatin) [34]. Similar to the pattern observed for mononucleosomes, H3.3 was depleted at the center of HCNEs, while being enriched at their borders and immediate flanking regions (Figure 2C and D).

Taken together, these findings indicate that HCNEs are characterized by a high nucleosome density, a low mononucleosome density, and a low H3.3 density in the S2 cell line. Thus, HCNEs appear to exist in a more compact chromatin environment compared to their flanking regions.

### Dynamic regulation of histone modification at HCNEs

Chemical modifications of histones can determine the state of chromatin and regulate gene expression [14]. Since HCNEs are believed to regulate developmental genes, we questioned whether their chromatin state might change during development. We tested six histone modification markers: H3 lysine 4 mono- and tri-methylation (H3K4me1 and H3K4me3), H3 lysine 9 acetylation and tri-methylation (H3K9ac and H3K9me3), and H3 lysine 27 acetylation and tri-methylation (H3K27ac and H3K27me3). In addition, we examined *nejire* (a CREB-binding protein; CBP) for any association with the regulation of transcription in our system.

H3K9me3 and H3K27me3, which have been associated with the repressed state of chromatin [40], were found to cover 19–45% of HCNEs throughout development (Table 1). This indicates that many HCNEs maintain a repressive chromatin state during the development of *D. melanogaster*. However, H3K9me3 and H3K27me3 had broad peaks that spanned many kb while the HCNEs covered only small parts of these regions (Figure S2). This suggests that the repressed chromatin state is a property of the regions that harbor HCNEs, and does not appear to be specific to HCNEs.

**Table 1:** Dynamic histone modification at HCNEs during *Drosophila* development.

	**Embryonic** **0–4** **hours**	**Embryonic** **4–8** **hours**	**Embryonic** **8–12** **hours**	**Embryonic** **12–16** **hours**	**Embryonic** **16–20** **hours**	**Embryonic** **20–24** **hours**	**Larva** **1**	**Larva** **2**	**Larva** **3**	**Pupa**	**Adult** **Male**	**Adult** **Female**
**CBP**	0.1	1.9	–	3.8	0	0.1	0.4	–	1.6	5.0	4.5	3.6
**H3K4me1**	7.0	0.7	9.3	25.3	14.1	2.8	0.3	3.4	0.5	3.2	0.1	1.3
**H3K4me3**	1.4	0.3	1.9	1.9	5.2	4.1	0	0.6	1.3	0	0.6	0.4
**H3K9ac**	5.6	2.7	5.8	3.4	5.1	7.6	0	1.3	1.4	0.3	0.3	0.8
**H3K9me3**	19.7	19.6	23.3	30.2	28.7	26.1	25.5	30.0	–	21.9	–	–
**H3K27ac**	5.3	3.8	4.7	10.9	6.3	4.4	0.4	23.2	3.8	2.1	0	2.7
**H3K27me3**	41.8	25.0	29.1	21.9	41.5	40.7	31.7	49.7	31.0	45.4	23.1	–

Table shows the percentage of HCNEs that were positive for any of the six analyzed histone modification markers or CBP binding across 12 developmental stages. A dash (−) indicates missing data. An HCNE is considered to have a given marker when at least 10% of its length overlapped with the marker. We observed a prominent presence of H3K27me3 and H3K9me3 among the HCNEs throughout development. Markers associated with the active chromatin state (H3K27ac, H3K9ac, and H3K4me3) displayed stage-specific patterns.

Although the histone modifications normally associated with active chromatin and CBP were relatively depleted among HCNEs (Table 1), they demonstrated significant stage-specific patterns. For example, H3K4me1 and H3K9ac were predominantly enriched during the embryonic stages, whereas the number of HCNEs with H3K27ac increased during the second larval stage and CBP was more abundant during later developmental stages (Table 1). Unlike the peaks seen for the repressive markers (see above), the peaks of these active markers were narrow, and thus appeared to reflect the activities of HCNEs rather than their surrounding regions (Figure S2).

### Association between the transcriptional activities of HCNE-proximal genes and histone modification at HCNEs

**Figure 3:**
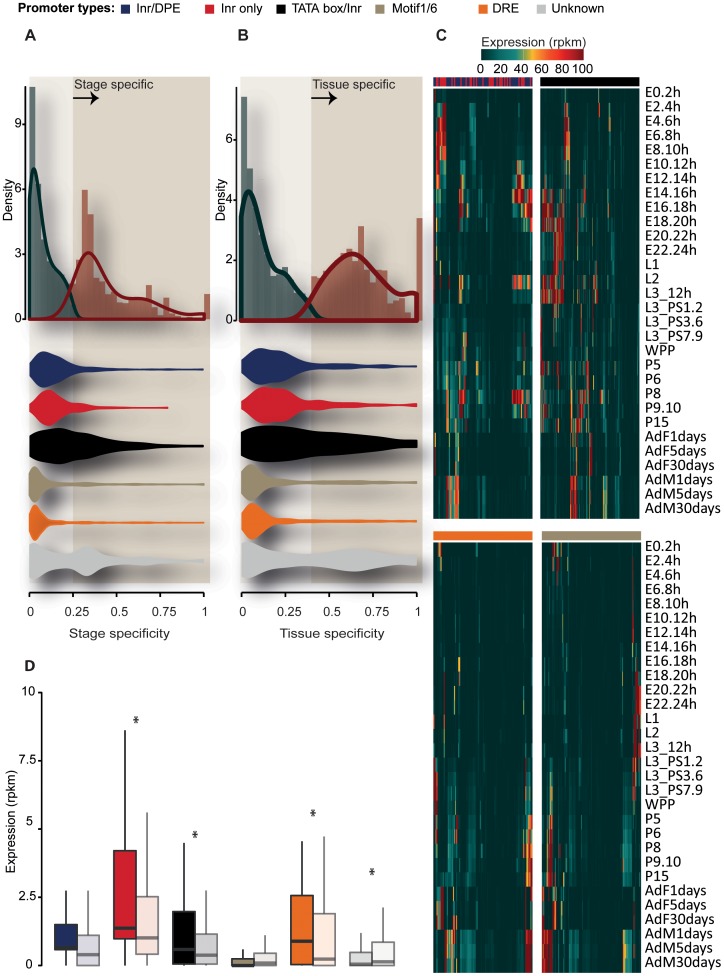
Transcriptional activity of HCNE-proximal genes and their associations with histone modifications of HCNEs. (A) (upper) Histogram showing the bimodal distribution of stage specificity amongst HCNE-proximal genes, measured as 1-entropy; 0 indicates that genes were expressed evenly across different stages, while 1 indicates that genes were expressed during only one stage. (lower) Violin plots showing the stage specificity of HCNE-proximal genes grouped by their core promoter type. Genes with Inr-motif promoters are more stage-specific than genes of the other core promoter types. (B) Same as in (A), but assessing tissue specificity. (C) Heatmaps illustrating the expression levels of stage-specific HCNE-proximal genes across 30 developmental stages (from FlyBase); E, L, P, AdF and AdM refer to Embryonic, Larva, Pupa, Adult Female and Adult Male stages. Genes were grouped by their promoter type (Color key for promoter type is shown on the top of each Heatmap; Inr only and Inr/DRE are grouped together for visualization purposes), and expression values greater than 100 were rounded to 100. Complete linkage hierarchical clustering is performed with Euclidean distance as the distance metric. Clusters of genes with Inr-motif promoters exhibit high levels of expression throughout development, whereas the genes having other promoter types are predominantly expressed during the later stages. (D) Boxplot showing differences in the expression levels of HCNE-proximal genes grouped by the presence (sharp color) or absence (faded color) of active markers at the nearest HCNEs. Expression levels were examined for all 12 developmental stages (from modEncode). Symbol ‘*’ indicates P<0.05.

We next examined the transcriptional activities of HCNE-proximal genes during 30 stages of development and across 28 different tissues. We first assessed their stage and tissue specificities with Shannon entropy (see Methods), and found that the stage and tissue specificities of HCNE-proximal gene expression followed a bimodal distribution (Figure 3A and B). This indicates that HCNEs are flanked by both stage/tissue-specific genes and those that are uniformly expressed across different stages and tissues. Moreover, we noted that genes with Inr-motif promoters exhibited higher degrees of stage- and tissue-specific expression compared to those with Motif1/6 and DRE promoters (Figure 3A and B, Table S5). This is consistent with our observations regarding the functional enrichment of genes of distinct promoter types (see above).

We next explored the expression profiles of the HCNE-proximal genes that showed stage/tissue-specific expression based on a stringent cutoff inferred from their bimodal distributions (1 - entropy ≤0.25 and 0.4 for stage and tissue specificity, respectively). Clusters of stage-specific genes with Inr-motif promoters showed high levels of expression across most developmental stages (Figure 3C). In contrast, genes with Motif1/6 and DRE promoters were predominantly expressed during later developmental stages. Interestingly, many of the selected genes with Motif1/6 or DRE promoters were male-specific (Figure 3C). Unlike the stage-specific genes, tissue-specific genes of different promoter types showed similar expression profiles across tissues. Interestingly, we observed large clusters of genes with Motif1/6 and DRE promoters expressed at high levels in ovary and testis (Figure S3).

Histone modifications around genes are often correlated with their transcriptional regulation. Thus, we studied whether histone modification and/or CBP binding at HCNEs could be associated with the transcription levels of nearby genes. For all promoter types, we grouped the stage-specific HCNE-proximal genes by the presence or absence of active markers (H3K4me1, H3K4me2, H3K9ac, H3K27ac, or CBP) at the nearest HCNE (A marker is considered present at HCNEs if at least 10% of HCNE length overlap with marker peak). Interestingly, genes with Inr only, TATA box/Inr and DRE promoters near active HCNEs showed higher expression levels than those near inactive HCNEs (P = 1.27e-11, P = 2.10e-2, and P = 7.64e-7, respectively; Figure 3D). This suggests that HCNEs and their histone modifications could be involved in the transcriptional regulation of adjacent genes.

### Some HCNEs are initiated early in replication

**Figure 4:**
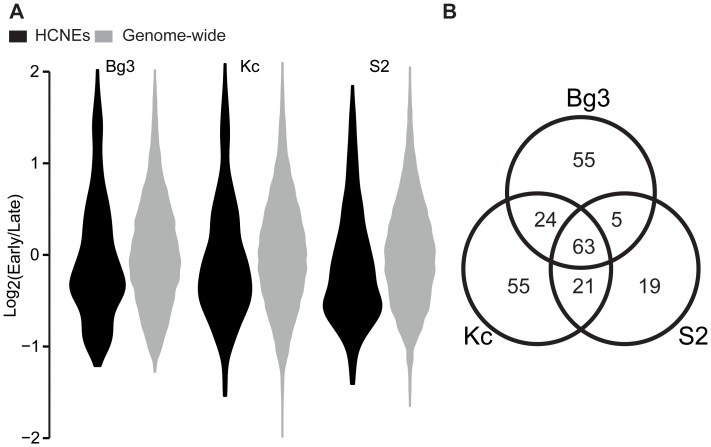
Some HCNEs fire early in replication. (A) Violin plot displaying the distributions of replication timing at HCNEs and other genomic regions in three cell lines. HCNEs fire later in replication compared to all genomic regions across all cell lines. (B) Venn diagram demonstrating the number of HCNEs within the early replication origin peaks identified in the three cell lines. Approximately 10% of HCNEs are associated with early replication origins; of them, only 26% are common to all three cell-lines, indicating that some HCNEs undergo cell-specific initiation of early replication.

DNA replication is a highly accurate process that ensures the correct transmission of genetic information to daughter cells. The location and temporal order of replication is conserved in several yeast species [41], but replication origins do not seem to be strongly conserved among higher eukaryotes [4]. Here, we examined the replication timing of HCNEs in three *D. melanogaster*-derived cell lines: ML-DmBG3-c2 (Bg3), Kc-167 (Kc), and S2-DRSC (S2) cells. We found that HCNEs fire later during replication compared to other genomic loci in Bg3 (P = 8.9e-9), Kc (P = 1.4e-9), and S2 (P = 5.45e-21) cells (Figure 4A). This result is consistent with the reported late replication of repressed and Polycomb-associated heterochromatic regions enriched in HCNEs [42]. However, we found a subset of HCNEs that serve as early replication origins, at percentages higher than those expected by chance: 10.09% (P = 7.7e-9), 11.20% (P = 1.4e-3) and 7.42% (P = 0.02) for Bg3, Kc and S2 cells, respectively (P-values computed by Fisher’s exact test). Only 26.03% of these early-replication HCNEs were common to all three cell lines (Figure 4B), indicating that the activities of HCNEs as early replication origins are cell-line-specific.

### Association of HCNEs with nuclear structures

The nuclear lamina, which is an important part of the nuclear structure, functions in important cellular processes of metazoan cells, including chromatin organization and DNA replication [43]. Several studies have identified lamina-associated domains (LADs) as genomic elements that are capable of mediating the association between the genome and the structural framework of the nucleus [44], [45]. We questioned whether the identified HCNEs could be associated with LADs. We downloaded the positions of LADs in the *D. melanogaster* genome [45] and converted the coordinates to those of the current genome release using the FlyBase conversion tool [46]. Consistent with the previous report that lamin protein binding is enhanced along HCNE-enriched repressed chromatin [42], we found that 872 HCNEs were located within LADs (P = 3.39e-32 by Fisher’s exact test). Our results therefore suggest that HCNEs can associate with structural components of the nucleus, potentially contributing to their evolutionary selection.

### Correlations among distinct properties of HCNEs

**Figure 5:**
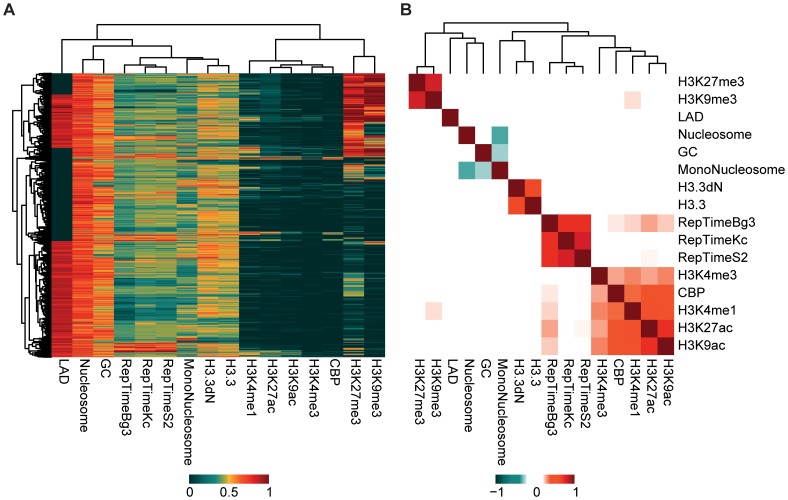
Genomic and epigenomic properties of HCNEs. (A) Heatmap demonstrating the clustering of HCNEs based on the studied features (see below). (B) Heatmap showing correlation between features. The studied features include: the levels of nucleosomes, mononucleosomes, H3.3 and H3.3dN; the summed occurrence of each histone modification and the CBP binding for each HCNE during development; the replication timing in the three studied cell lines (columns labeled as RepTimeBg3, RepTimeS2 and RepTimeKc); and the LAD scores. To facilitate visualization, the values of each feature were scaled to between 0 and 1 using the equation(*value – min)/(max – min)*, where *max* and *min* were the maximum and minimum values of each feature, respectively; complete linkage hierarchical clustering is performed with Euclidean distance as the distance metric.

To gain new insights into the overall properties of HCNEs, we performed cluster analysis (complete-linkage hierarchical clustering) using the identified HCNEs and the examined genomic and epigenomic features (Figure 5 and Table S6). To reduce the complexity of this analysis, we summed the number of histone modifications and CBP bindings across the various developmental stages.

A number of HCNE clusters were observed. The members of the most prominent cluster were tri-methylated on H3 during development (H3K9me3 and H3K27me3; dark red cells in the bottom and middle clusters of Figures 5A). As these markers have been associated with transcriptional repression [40], our results may indicate that these HCNEs are silenced throughout most of development. Although the activation markers (H3K4me1, H3K4me3, H3K9ac, H3K27ac, and CBP) [40] were generally depleted on HCNEs, we observed a few small clusters of HCNEs that exhibited various combinations of activation modifications throughout development. This suggests that although HCNEs were typically maintained in a repressed state in the studied cell lines and during most developmental stages, they may be activated in specific cell types or for short periods during development. Similar patterns have been observed among some developmental genes [47].

Other HCNEs were found to be associated with early replication origins (Figures 5A, dark red cells in columns 4, 5 and 6). This suggests that replication may be tightly connected with the mechanisms underlying the genomic conservation of some HCNEs.

Our correlation analysis further revealed the following relationships at HCNE (Figures 5B): a negative correlation between mononucleosome levels and GC content/nucleosome occupancy; a positive correlation between H3.3 and H3.3dN; a positive correlation between the repression markers, H3K9me3 and H3K27me3; positive correlations among the activation markers, CBP, H3K9ac, H3K4me1 and H3K27ac; and positive correlations among replication origins. Weak positive correlations were also observed between replication markers and activation markers (Figures 5B). These correlations between the different features indicate that they all act on roughly the same subsets of HCNEs. However, this held true only between features from a given cell line or developmental stage. HCNEs appeared to be mostly repressed in S2 cells, while being active during some of the studied developmental stages of the fly. Thus, nucleosome occupancy and other chromatin features from the S2 cell line do not appear to correlate with the histone modification marker data from the various developmental stages.

## Discussion

### Local trends in GC and nucleosome density provide insights into DNA conservation

In HCNEs, elevated GC levels and associations with developmental genes appear to be universal beyond the kingdom level [4], [22]. Recent studies have suggested that compensatory evolution may exist, as divergent sequences have been found to maintain their GC contents and nucleosomal organizations in yeast [48]. Short conserved elements (30–70 bp) in *D. melanogaster* also showed similar patterns [22]. Local GC contents have been found to exert strong effects on the flexible organization of nucleosome spacing, with AT-rich sequences serving as repelling elements and forcing the nucleosome to position on GC-rich areas that favor nucleosome binding. Nucleosomes suppress C to T, G to T, and A to T mutations by about 2-fold in yeast by reducing the exposure of naked DNA [15]. These observations are consistent with our findings that HCNEs are GC- and nucleosome-rich, and are demarcated by AT- and mononucleosome-rich sequences (Figures 1 and 2).

The nucleosome data used in this study were obtained from S2 cells, which originated from a late embryonic macrophage-like lineage [49]. Our results suggest that nucleosomes remain dormant on HCNEs, at least in S2 cells. These results may be cell-specific, however, as nucleosome occupancy can differ in various situations, including during development [50].

### Histone modifications suggest that HCNEs may play a regulatory role

Chromatin is not a static entity. Chemical and thermal fluctuations around chromatin can denature the DNA (i.e., the so-called “DNA breathing”) [15], and dynamic changes can occur via histone modifications. Most of the previous studies of epigenetic regulation have focused on genes and promoter regions with well-defined locations and properties. The epigenetic regulation mechanisms of other locations, such as distal enhancers, are not yet well understood because it is difficult to identify such elements and their long-distance relationships with target genes. Here, we examined possible epigenetic control mechanisms at HCNEs, and identified their dominant histone modifications, which included H3K9me3 and H3K27me3 (Table 1).

Histone modification is often associated with the transcriptional activation or repression of nearby target genes. Consistent with this, many HCNE-proximal genes, most notably genes with Inr-motif and DRE promoters, showed higher expression levels when their associated HCNEs were in the active chromatin state (Figure 3D). This suggests that the chromatin modification of HCNEs may modulate the transcription of those genes, many of which are developmental regulators. *Cis*-regulation can control distant genes, but it is generally difficult to discriminate between target and bystander genes due to the lack of comprehensive transcriptomic and epigenetic data [16] and the complications caused by high gene density, such as that found in the *D. melanogaster* genome.

### Replication timing at HCNEs

The orchestrated and properly timed initiation of replication from multiple origins during cell division is essential for the vertical transfer of genetic materials. Thus, most species maintain common genomic and epigenomic features at their replication origins. Replication origins are GC-rich, but their nascent strands are AT-rich, allowing the DNA to open easily [51]. In addition, they are lineage-specific, and do not appear to be related to the conservation level of DNA in higher eukaryotes [4], [52]. Replication origins fire at different times, and early replication origins tend to allow for fewer mutations in cancer cells [53]. Our results indicate that most HCNEs initiate late in replication. However, about 10% of HCNEs co-localize with early replication start sites, at least in *D. melanogaster*, and thus should be under strong negative selection. Future work is needed to identify the molecular mechanism(s) through which the origin-recognition complex recognizes these specific sequences.

### Crosstalk between HCNEs and the nuclear architecture

Previous studies have shown that HCNEs, including ultraconserved elements, are not mutational cold spots [4], [54], [55], suggesting that they are likely to have intrinsically important functions and be under strong selection pressures. However, it is unlikely that every base of a ≥200 bp HCNE plays a regulatory role, suggesting other vital functions are likely to be involved in the negative selection of these elements.

Studies have shown that genomic elements and nuclear structures frequently undergo crosstalk and dynamic regulation. The nuclear scaffold/matrix provides both a mechanical anchor and distinct territories for genomic elements and proteins during various processes, such as replication and transcription [56]. Although we found some overlap between HCNEs and S/MARs, the amount of overlap was not statistically significant (data not shown). This could reflect the generally poor identification of S/MARs in insects; only a very small number of S/MARs have been experimentally verified, and the computational prediction of such regions has been far from accurate due to their sequence diversity [57]. However, overlap between HCNEs and S/MARs has been reported in other lineages [18], [58], and these elements have some common characteristics (e.g., associations with developmental genes), seeming to indicate that they may interact.

LADs have been relatively well characterized in the *D. melanogaster* genome [45], which is ∼40% covered by LADs of varying size (7∼700 kb). LADs are closely related to S/MARs and significantly overlap with S/MARs in human cells [56]. Lamin B_1_, which is the primary component of the nuclear lamina, binds to matrix-attachment regions [59]. Lamin also binds histones and is involved in chromatin remodeling, DNA replication, apoptosis, and early development [43]. Thus, lamin could arguably link the genome to nuclear structures. We observed a striking overlap between LADs and HCNEs indicating possible intimate relationship between the nuclear lamina and mechanisms of genomic conservation. The LAD data used in the present study were obtained from *Drosophila* Kc cells [45]. The composition of the nuclear lamina changes during development; for example, lamin B_1_ predominates in the early chicken embryo and decreases thereafter, whereas lamin A shows the opposite expression pattern [60]. In the future, it will be interesting to examine the interaction between conserved elements and various lamin proteins during development and in different cell types. Lamins are exclusive to metazoan cells, and are not detected in plant cells (reviewed in [43], [61]). Thus, although HCNEs have similar properties in animals and plants, it is likely that different nuclear proteins may be involved in their structural associations with the nuclear matrix in these two systems.

Because HCNEs do not represent mutational cold spots [4], [54], [55], any mutations in these elements must be repaired. Very little is known regarding the binding preferences and recruitment mechanisms of DNA-repair proteins. A few lines of evidence have indicated that DNA repair-related proteins show weak sequence preferences in other species [62], [63]. However, additional detailed molecular studies will be required to assess the repair mechanisms that may be responsible for suppressing mutations in HCNEs. We also need future studies of nucleosome territories [64] to understand how these repair proteins gain easy access to the mutated region. Multifunctional and highly conserved chromatin-related proteins could be considered as candidate regulators for this mechanism. One such protein is Heterochromatin Protein 1 (HP1), which binds to the nuclear envelope, histones, replication origins, and DNA-damage-response proteins [51], [65]–[67].

## Materials and Methods

### Extraction of HCNEs and generation of background sequences

We downloaded the DM3 compilation of the *D. melanogaster* genome (Apr. 2006, BDGP Release 5) and extracted highly conserved elements using the ECE algorithm (https://github.com/Magdoll//ECE) [68] from the phastCons score tracks of 14 insect genomes, as obtained from the UCSC Genome Browser [69], [70]. We set the minimum length and the phastCons conservation score to 200 bp and 0.95, respectively. We trimmed highly conserved elements overlapping coding regions based on the R5.46 genome annotation from FlyBase [71], and filtered out elements shorter than 200 bp. We used BEDTools [72] to extract HCNE sequences and 10,000 random noncoding sequences having the same length distribution and the same distance distribution to the nearest gene or exon, compared to the intergenic and intronic HCNEs, respectively.

### Analysis of HCNE sequence properties

To investigate possible TFBSs within HCNEs, we identified overrepresented heptamers in HCNEs sequences and queried the top 50 against FlyReg motifs [23] using STAMP [24]. To identify overrepresented heptamers, we computed the binomial probability of whether their observed frequency in HCNEs is higher than expected by chance. We estimated the expected frequency of a heptamer by averaging its frequencies over 10,000 datasets sampled from random noncoding backgrounds (described above); each dataset contains same number of sequences as HCNEs.

### Identification of HCNE-proximal genes

We downloaded a list of 1,321 genes showing conserved microsynteny in the *Drosophila* genus, along with their microsyntenic blocks mapping [28]. We used FlyMine [73] to obtain the gene coordinates, and found that two of them were absent from current genome annotation. We determined the coordinates of the GRBs based on the coordinates of genes located at the boundaries of the microsyntenic blocks. For downstream analysis, we selected all genes within 50 kb from GRB boundaries plus genes within 50 kb from boundaries of HCNEs that were not near a GRB.

To determine the promoter type of the HCNE-proximal genes, we download McPromoter [30] predictions from the current genome release [http://tools.genome.duke.edu/generegulation/McPromoter006/mcpromoter.rel5.thres0.03.gff], using a stringent threshold of 0.03. Each gene was assigned the promoter type prediction found within 500 bp upstream of the TSS and a minimum of 500 bp and the length of 5′UTR downstream of the TSS. When multiple predictions were made for a given gene, we chose the one with the highest score. The 5′UTR coordinates were obtained from FlyMine [73], which was also used to compute the protein domains and assess GO enrichment.

### Epigenomic data

We obtained the preprocessed nucleosome occupancy data (profiling by genome-wide tiling array) deposited by Henikoff et al. [33] from the NCBI GEO database (GSE13217): the nucleosome densities were the averages obtained from GSM333835, GSM333840, and GSM333844; the mononucleosome data were the averages obtained from GSM333837 and GSM333841; the H3.3 occupancies were obtained from GSM333869; and the H3.3dN occupancies were taken from GSM333870. The data for the six studied histone modification markers [H3 lysine 4 mono- and tri-methylation (H3K4me1 and H3K4me3), H3 lysine 9 acetylation and tri-methylation (H3K9ac and H3K9me3) and H3 lysine 27 acetylation and tri-methylation (H3K27ac and H3K27me3)], CBP binding, and replication time were obtained from the modENCODE project. These data span 12 developmental stages, including six embryonic stages, three larval stages, and the pupae, adult male, and adult female. The data were downloaded from the online server [ftp://data.modencode.org/D.melanogaster/Histone-Modification/ChIP-seq/computed-peaks_gff3/]. Some developmental time points were missing for some of the histone markers. For example, the larva 3, adult male and adult female stages were missing data for H3K9me3 and the adult female stage was missing data for H3K27me3. We obtained ChIP-Seq peaks for CBPs throughout the same developmental stages, with the exceptions of the embryonic 8 to 12 h and larva 2 stages, which were missing. Missing data were ignored in our analysis. The histone modification and CBP binding data can be found in the modENCODE depository under the following IDs (DCCids): modENCODE_862, modENCODE_863, modENCODE_854, modENCODE_856, modENCODE_857 modENCODE_855, modENCODE_859, modENCODE_860, modENCODE_861, and modENCODE_858. The data for our genome-wide replication timing characterization and the early origin of replication peaks can be found under the following DCCids: modENCODE_668, modENCODE_66 and modENCODE_670 (for replication timing) and modENCODE_3441 (for the early origin of replication peaks).

Due to the lack of raw data for many of the studied epigenomic modifications (which is required for normalization across conditions), we analyzed the frequencies of the histone modification ChIP-Seq peaks on HCNEs rather than the enrichment levels of these modifications. We considered a marker present in an HCNE if the overlap between the peak and HCNE covered at least 10% of the HCNE length.

### Gene expression data and analysis

We downloaded the normalized read counts of *D. melanogaster* in reads per kilobase per million (RPKM) for 30 developmental stages and 28 tissues, as compiled in FlyBase. The utilized data are available at FlyBase [ftp://ftp.flybase.net/releases/FB2014_02/precomputed_files/genes/gene_rpkm_report_fb_2014_02.tsv.gz]. We changed values greater than 100 to 100 (i.e., they were considered to be very highly expressed).

To assess the relationship between histone modification at HCNEs and the transcriptional activity of proximal genes, we downloaded files of aligned reads from the modENCODE ftp server [ftp://data.modencode.org/D.melanogaster/mRNA/RNA-seq/alignment_sam/]. We computed normalized read counts (in RPKM) for each gene.

We computed the Shannon entropy for the expression of each gene using the following formula:
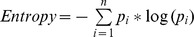



Where 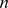 is the number of conditions; and 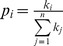, where 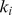 is the gene expression level at condition 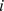. We used *n* as the base of the log in order to keep the value between 0 and 1. Genes with uniform distribution of expression had entropy of 1, while those expressed under only one condition had entropy of 0.

## Supporting Information

Figure S1 PhastCons score distributions for exons, intergenic regions, and introns.
(TIF)

Figure S2 Boxplot illustrates HCNE coverage for various histone modifications and CBP peaks.
(TIF)

Figure S3 Heatmaps illustrating the expression levels of tissue-specific HCNE proximal genes across 28 different tissues (similar to Figure 3C).
(TIF)

Table S1 Table listing the HCNEs defined in this study.
(XLSX)

Table S2 Table mapping the top 50 overrepresented heptamers to known transcription factor binding sites.
(XLSX)

Table S3 Table listing the Gene Ontology enrichment (biological processes only) among the HCNE-proximal genes.
(XLSX)

Table S4 Table listing the protein domains found to be enriched among the HCNE-proximal genes.
(XLSX)

Table S5 Table listing P-values associated with Figure 3A–B. P-values obtained by comparing the stage and tissue specificity distributions between genes of different core promoter types.
(XLSX)

Table S6 Table contains the values of the analyzed features used in the clustering analysis (Figure 6).
(XLSX)
