# Supplementary figures and images for: Dynamic Epigenetic Control of Highly Conserved Noncoding Elements
Source: PLoS One. 2014 Oct 7;9(10):e109326. doi: 10.1371/journal.pone.0109326 (PMC4188601; doi:10.1371/journal.pone.0109326)

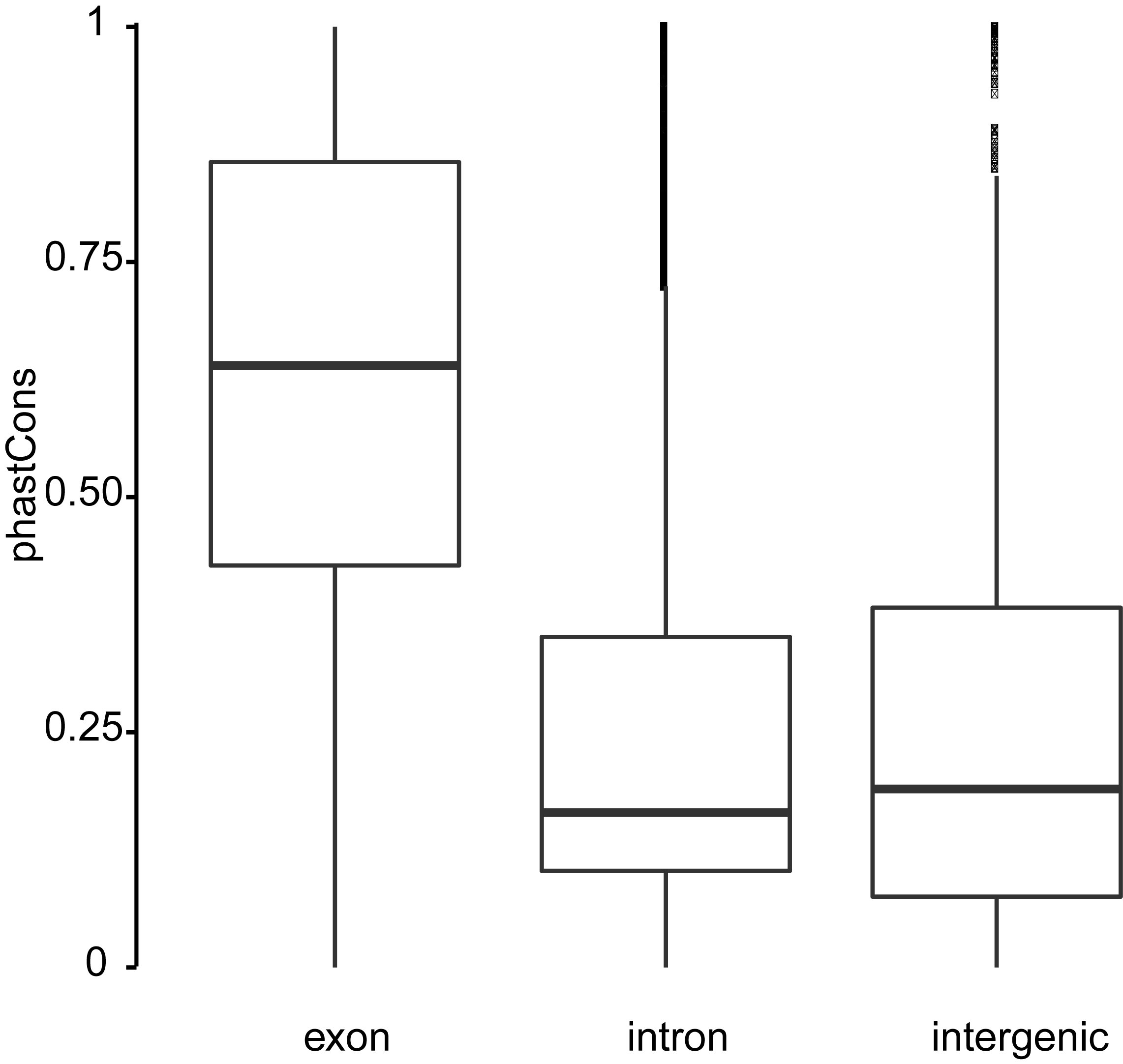

Supplement: Figure S1 — PhastCons score distributions for exons, intergenic regions, and introns. (TIF) [file pone.0109326.s001.tif]

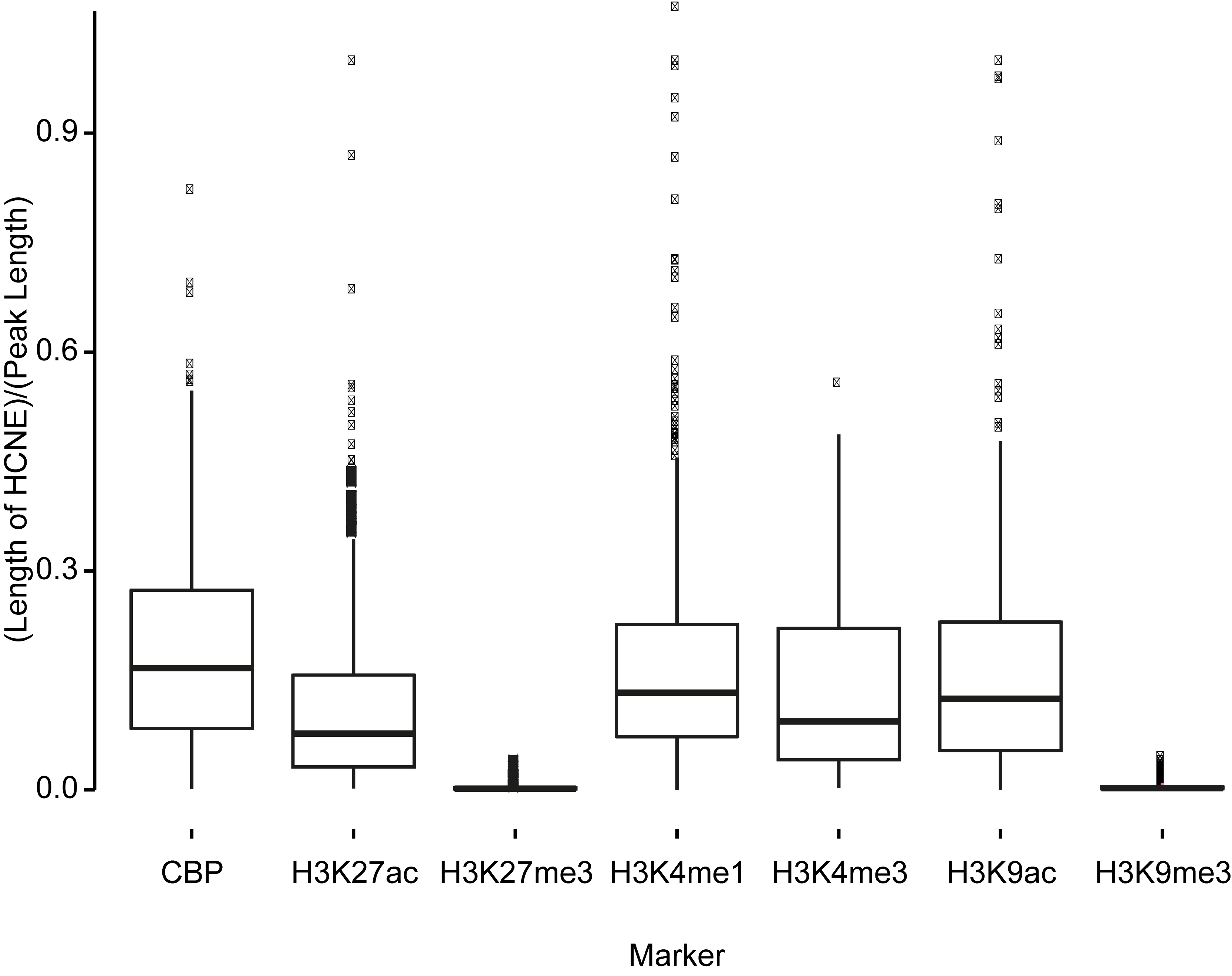

Supplement: Figure S2 — Boxplot illustrates HCNE coverage for various histone modifications and CBP peaks. (TIF) [file pone.0109326.s002.tif]

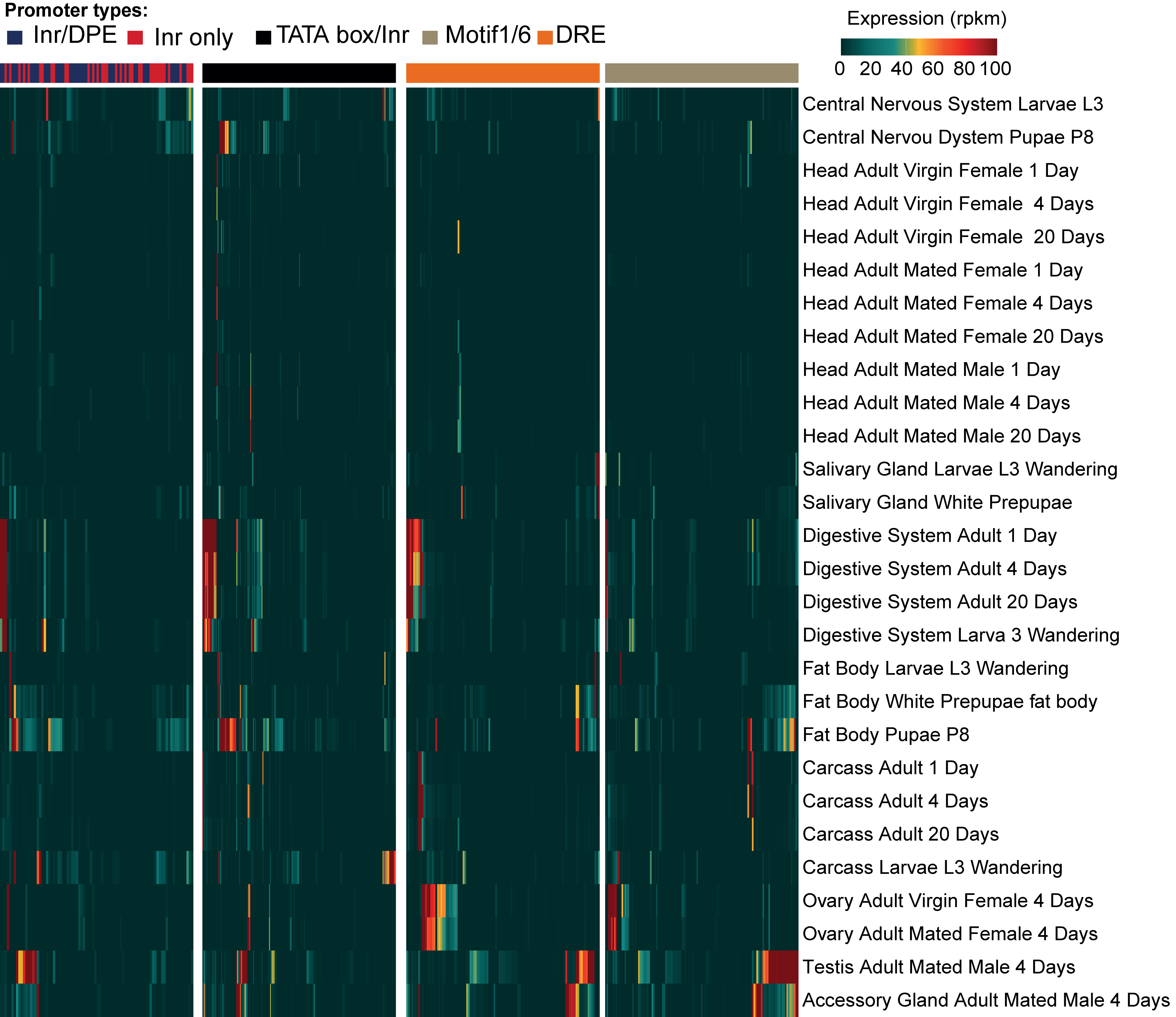

Supplement: Figure S3 — Heatmaps illustrating the expression levels of tissue-specific HCNE proximal genes across 28 different tissues (similar to Figure 3C). (TIF) [file pone.0109326.s003.tif]
